# Work in progress, developing of guidelines - How could EPMA contribute to better health care in developing countries

**DOI:** 10.1186/1878-5085-5-S1-A10

**Published:** 2014-02-11

**Authors:** Anna Benini

**Affiliations:** 1 UNO, Copenhagen, Denmark

## Introduction and objectives

Predictive preventive and personalized medicine is certainly far from optimal also in industrialized countries, in particular as far as the predictive aspects, but also the preventive ones. In fact the predictive aspects are still a sort of goal and final scope also in countries with the “best” medical care!

The level of development of medical care in industrialized countries is various and depends to a certain level on each country, but it might be generally considered to be at “reasonable good” level, although certainly not optimal, taking into account present economical constrains. Therefore it is sometimes a short of a shock to work on projects in developing countries. In my case, as I have been experiencing a lot of changes in direction in medical health care. It comes natural to look back at try to analyze different tendencies, also related to economical situations. From the period of building big hospitals and medical structures to the nowadays tendency to reduce the stay of patients to absolute minimum and other examples. Difficult to say what is the best approach as diagnostic and therapeutically means have changed so much due to fast technical as well as clinical developments. Now comes a big question: is there a short cut for developing countries? In case it might be possible to go back 30-40 years ago, what could be done to reach a good level of health care, better than present, with more prevention, avoiding the mistakes and focusing on the right objectives. The role of errors and trials is certainly important but that has already been experienced and therefore it should be a must to take advantage of other experiences to evaluate pros and cons and better understand priorities and objectives. The possibility “to go back in time” is everybody’s dream, and, it is what (to a certain extent) could happen in developing countries. In fact, in many countries the level of health care is more or less comparable to our realities 30- 50 years ago (clearly depending on the country). This is a challenging opportunity indeed! It is a big responsibility to let it go in vane! But, unfortunately, from my experience, this is what is happening in many developing countries, starting a fast as well as useless “competition” with industrialized countries. It might look more important to buy some top “high tec” equipment than invest on basic medical structures, education and prevention.

## Practical approach: creation of working groups

For these reasons, I believe the role of EPMA might be crucial. A while ago I submitted this idea to EPMA responsible Officers and I am glad that it was considered in a positive way. I have started with the organization of two working groups, the first on cancer prevention and recently a second one on cardiovascular diseases. My approach being to evaluate what available and possibly already into practice, in different countries and than select the participants depending first on the quality of the project, feasibility and results; taking into account their availability to participate in the working group.

### 1) Cancer prevention

The prevention program in Peru’ together with the one in Tanzania has been selected. Prof Dino Amadori, head of IRST (Istituto Scientifico Romagnolo per Studio e Cura Tumori) in Italy, is supporting the program in Tanzania, while Dr. Gustavo Sarria is responsible for the national cancer prevention program in Peru’. Both programs have reached impressive results.

### 2) Cardiovascular diseases

Despites of the very difficult condition of the country, there are considerable effort to start a prevention program of cardiovascular diseases in the rural part of Nepal. The effort is remarkable. Due to previous collaboration, I have put together the work of Dr. Rabi Mala from the Heart Center in Katmandu with Dr. Erik Jorgensen of the Copenhagen University Hospital.

A preliminary evaluation of the work of these two groups will be presented on the Congress. Other working groups are in the pipeline. In particular the ENT working group is about to start. It is in the intention of the working groups, to also develop a feasibility scheme for the application of the guidelines including cost and investments.

## Conclusions

It is well known that even from the economical point of view prevention is by far much “cheaper and more effective” than treatment, and of course it gives a much better quality of life. Therefore it is painful to see that in countries where there is the possibility to go “back in time”, the main concern seems to be to make better and faster the mistakes done in industrialized countries! The role of EPMA in this respect is crucial in promoting working groups and guidelines about how to appropriately develop preventive as well as treatment structures; promote campaigns of education and sensibilization of the people about basic preventive rules, early detection and identification of problems and medical symptoms. At the same time it is also important to put emphasis about prevention at international levels in order that it should be equally “appealing” for developing countries to invest in prevention structures also when there is less sophisticated equipment involved.

